# *BRCA1/2* Mutation Types Do Not Affect Prognosis in Ovarian Cancer Patients

**DOI:** 10.3390/curroncol28060377

**Published:** 2021-11-03

**Authors:** Michalis Liontos, Eleni Zografos, Panagiotis Zoumpourlis, Angeliki Andrikopoulou, Anna Svarna, Oraianthi Fiste, Elena Kunadis, Alkistis Maria Papatheodoridi, Maria Kaparelou, Konstantinos Koutsoukos, Nikoloas Thomakos, Dimitrios Haidopoulos, Alexandros Rodolakis, Meletios-Athanasios Dimopoulos, Flora Zagouri

**Affiliations:** 1 Oncology Unit, Department of Clinical Therapeutics, Alexandra Hospital, National and Kapodistrian University of Athens, 11528 Athens, Greece; elzogra@med.uoa.gr (E.Z.); panos_zoubourlis@hotmail.com (P.Z.); aggandrikop@med.uoa.gr (A.A.); anna.svarna@hotmail.com (A.S.); ofiste@med.uoa.gr (O.F.); ekunadis@med.uoa.gr (E.K.); alkipapath@med.uoa.gr (A.M.P.); mkaparelou@yahoo.com (M.K.); koutsoukos.k@gmail.com (K.K.); mdimop@med.uoa.gr (M.-A.D.); florazagouri@yahoo.co.uk (F.Z.); 2 1st Department of Obstetrics and Gyencology, Alexandra Hospital, National and Kapodistrian University of Athens, 11528 Athens, Greece; thomakir@hotmail.com (N.T.); dhaidopoulos@hotmail.com (D.H.); arodolak@med.uoa.gr (A.R.)

**Keywords:** ovarian cancer, *BRCA*, germline mutations, survival

## Abstract

Background: High grade serous ovarian carcinoma (HGSOC) is the most lethal type of epithelial ovarian cancer, with a prevalence of germline *BRCA1/2* mutations as high as 20%. Our objective is to determine whether the location of mutations in the different domains of the *BRCA1/2* genes affects the clinical outcome of HGSOC patients. Methods: A total of 51 women with *BRCA1* or *BRCA2* mutated ovarian cancer were identified. Progression-free survival (PFS) and overall survival (OS) were analyzed. Results: In our study cohort, 35 patients were carriers of germline mutations in *BRCA1* and 16 in *BRCA2*. The median PFS time following completion of the primary therapy was 23.8 months (95% CI 20.1–27.5) and the median OS was 92.9 months (95% CI 69.8–116.1) in all *BRCA* carriers. After multivariate analysis, no significant association among the location or type of *BRCA1/2* mutation with PFS or OS was identified. Notably, significant differences in PFS between carriers of identical mutations in the same *BRCA* gene were detected. Conclusions: Among HGSOC patients, *BRCA1/2* carriers with mutations in different locations of the genes show no significant difference in survival outcomes, in terms of PFS and OS, suggesting the potential effect of other genetic abnormalities and co-contributing risk factors.

## 1. Introduction

Despite advances in therapy, ovarian cancer remains a major health concern worldwide, with an estimated 313,959 new cases predicted for 2020 [1]. Many of these cases have been attributed to germline mutations in *BRCA1* and *BRCA2* genes, which is a frequent molecular event in women with the most lethal and prevalent type of epithelial ovarian cancer, high grade serous ovarian carcinoma (HGSOC) [2]. More specifically, the prevalence of germline *BRCA1/2* mutations in patients with this histology is up to 20%, while an additional 5–10% bear tumors with somatic mutations [3]. This information has important clinical implications since individuals with deleterious *BRCA* mutations compared to non-carriers are known to have superior prognosis [2], exhibit better responses to platinum-based chemotherapy [4], and derive greater benefit from PARP inhibition treatment either in the frontline [3,5,6], or in the platinum-sensitive relapse setting [7,8,9].

The genetic basis for ovarian cancer predisposition was originally established with the identification of *BRCA1* (OMIM# 113705) and *BRCA2* (OMIM# 600185), which are both tumor suppressor genes implicated in the mechanism of Homologous Recombination (HR) repair of DNA double-strand breaks [10]. However, despite the phenotypic similarities induced by *BRCA1* or *BRCA2* gene disruption, the proteins encoded by these genes are involved in different macromolecular complexes and have distinct biological roles in the regulation of the HR mechanism [11]. On the one hand, each functional domain of BRCA1 interacts with tumor suppressors, DNA repair proteins and cell cycle regulators to activate DNA damage checkpoints and repair, including but not limited to HR [12]. On the other hand, BRCA2 is not a versatile protein since its activity mainly involves regulation of RAD51, which is required for HR repair [13]. The different roles of *BRCA1* and *BRCA2* in genome protection confer distinct breast and ovarian cancer predisposition in mutation carriers [11]. Specifically, results of previous studies suggest that patients with high grade serous ovarian cancer and either *BRCA1* or *BRCA2* mutations have different clinicopathological features, response to treatment, and prognosis [14,15]. However, the etiology of why some *BRCA* carriers fare better than others is unclear.

To date, little is known about the clinical impact of differently mutated *BRCA1/BRCA2* domains on high grade serous ovarian cancer prognosis. In fact, both *BRCA* genes have complex genomic structures and encode relatively large proteins that are organized into distinct structural domains, each of which has selected binding partners and thus differential functions [16]. The *BRCA1* gene consists of 24 exons and the *BRCA2* of 27 exons, coding for protein products of 1863 and 3418 amino acids, respectively. *BRCA1* is composed of the zinc-binding N-terminal RING finger domain that is recognized as a ubiquitin E3 ligase enzyme, and the phosphoprotein-binding C-terminal BRCT domain, which is a transcriptional activation region that contributes to DNA repair [17]. Notably, exons 11–13 encode modular protein domains that act as binding sites for various macromolecules, such as RB, PALB2, c-Myc, RAD50,51 [17]. Concerning *BRCA2*, it encompasses a large DNA-binding domain (DBD) and eight copies of a 20–30 amino acid motif, known as BRC repeats, that bind RAD51 to regulate HR repair of DNA [18]. More than 2200 *BRCA1* and 2600 *BRCA2* pathogenic germline variants have been reported in the ClinVar database, maintained at the National Institutes of Health (NIH). The mutations reported so far are scattered throughout the genomic sequence of both genes and affect their structural and functional integrity in a different manner.

There has been limited previous evidence on the effects of cancer-causing *BRCA1/2* gene mutations located in different domains of the molecules on the prognosis of HGSOC patients. Thus, the current report aimed to evaluate the correlation between location of mutation within the *BRCA* genes and clinical outcome, in the context of progression-free survival (PFS) and overall survival (OS), in high grade serous ovarian cancer patients.

## 2. Results

### 2.1. Characteristics of the Study Cohort

Fifty-one patients with high grade serous ovarian carcinoma that bared germline mutations in the *BRCA1/2* genes were included in this study. The clinical, genetic and treatment characteristics of these patients are summarized in Table 1. More specifically, the median age of the patients was 54.2 years. Most patients presented at advanced stages (III/IV: 84.4%). Concerning the surgical management, 60.8% of the patients underwent primary debulking surgery and the remaining had interval cytoreductive surgery. The outcome of the surgical intervention was complete debulking (no residual disease) in 45 patients (88.2%). Notably, 39.2% of ovarian cancer patients received bevacizumab and in 66.7% of cases a PARP inhibitor was administered.

### 2.2. Location of Mutations in BRCA and Survival in the Study Cohort

Among the 51 participants of the study cohort, 35 were found to carry a *BRCA1* mutation and 16 patients a *BRCA2* mutation. Over half of the detected mutations were deletions (51.0%), while 33.3% of our group of patients harbored missense mutations. Concerning the *BRCA1*-mutated ovarian cancers, an equal percentage of mutations were located either within exons 11–13 (45.7%) or in the BRCT domain (45.7%). Among the 16 *BRCA2* carriers of the study cohort, 11 (68.8%) had mutations located within the RAD51-binding domain of the gene.

**Figure 1:**
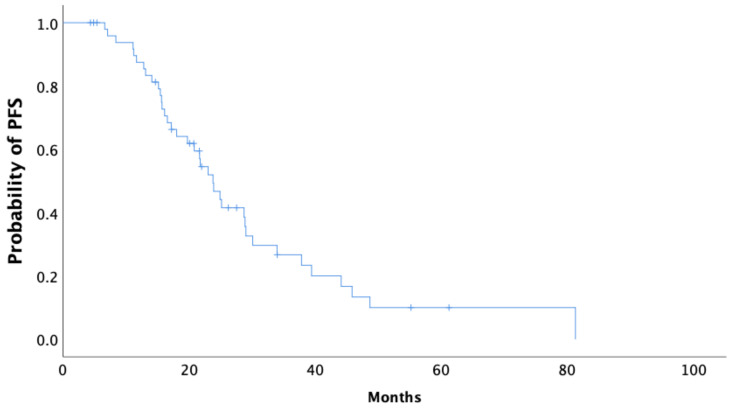
Kaplan–Meier curve of progression-free survival (PFS) of all *BRCA1/2* carriers.

**Figure 2:**
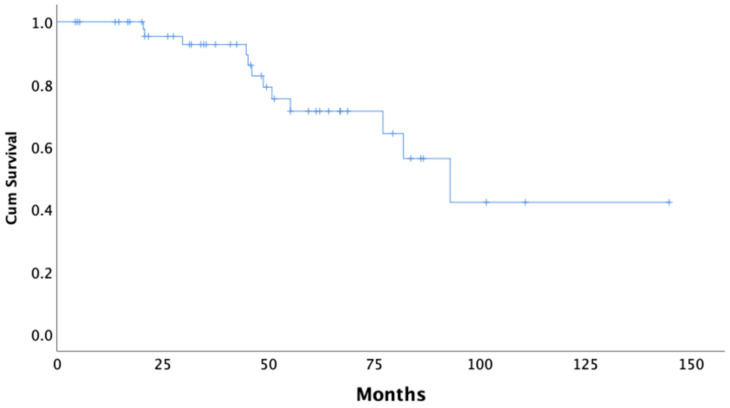
Kaplan–Meier curve of overall survival (OS) of all *BRCA1/2* carriers.

Median follow-up of the *BRCA1/2* mutation carriers was 45.8 months. In our cohort, the median time to the first recurrence (PFS) of disease following completion of the primary therapy was 23.8 months (95% CI 20.1–27.5) (Figure 1). The median OS from the date of diagnosis was 92.9 months (95% CI 69.8–116.1) in all *BRCA* carriers, as is shown using Kaplan–Meier survival estimates (Figure 2).

The univariate analysis showed that there was no significant association of the location or type of *BRCA1/2* mutation with PFS or OS duration (Table 2). Despite numerical differences, pairwise comparisons between different functional domains of each gene also did not indicate any significant difference in either PFS or OS (Figure S1). The only characteristic that showed a correlation with survival outcome was surgical outcome, with patients having no residual disease following debulking surgery exhibiting a significantly higher PFS (*p* < 0.001) and OS (*p* = 0.028) compared to those with optimal or suboptimal surgical results (Table 2).

**Figure 3:**
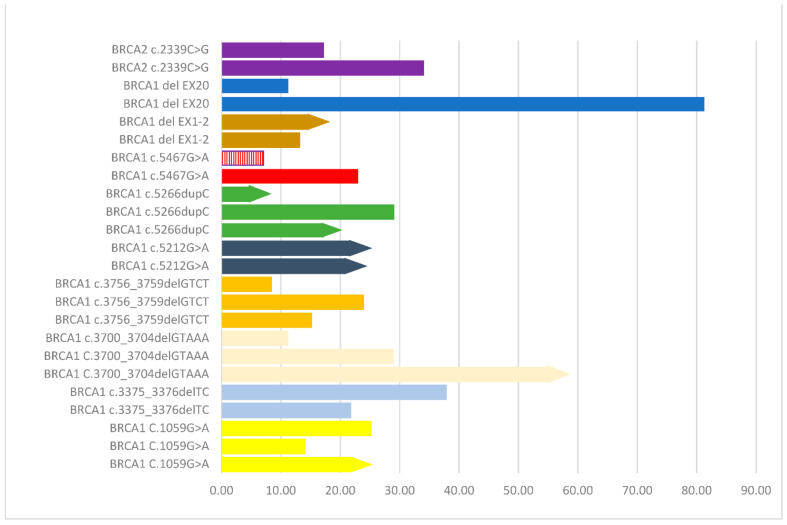
Analysis of progression free survival (PFS) among patients harboring the same mutation in the same BRCA gene. Identical mutations are depicted with the same color. Bars with an arrow at the end of them indicate no disease progression. The bar in red and white stripes indicates a case that received a PARP inhibitor as first-line therapy.

We further explored the effect of individual mutations that were common among patients in our cohort. Ten individual mutations were common among two or more patients. The results of the analysis of PFS among patients harboring the same mutation are graphically presented in Figure 3, showing noteworthy differences between carriers of identical mutations in the same *BRCA* gene.

## 3. Discussion

The *BRCA1* and *BRCA2* gene status undisputedly plays a key role in genome integrity and hereditary ovarian cancer pathogenesis; however, the frequency and precise prognostic role of *BRCA* mutations at different locations of the genes remains largely unknown. In this study, we addressed in a series of 51 high grade serous ovarian cancer (HGSOC) patients the correlation between *BRCA1/2* genotype and their survival, in accordance with the functional regions of the proteins. Our results demonstrated that *BRCA1* mutations were mainly located either in exons 11–13 (16/35), which encode modular protein binding domains, or in the BRCT domain (16/35). Additionally, among ovarian cancer patients harboring *BRCA2* mutations, most of them were located at exon 11 (11/16), affecting the RAD51-binding domain of the protein. Despite these noteworthy observations, data from our cohort of patients showed that mutations occurring at different locations of the *BRCA1* and *BRCA2* genes did not impact the survival outcome, in terms of PFS and OS. Additionally, those treated with PARP inhibitors, which carried distinct germline mutations located at different domains of the *BRCA1/2* genes, exhibit no difference in survival among each other. These are important findings in the understanding of the genomic background of *BRCA*-mutated HGSOC and their implications are discussed in detail in the following section.

Several lines of evidence indicate that specific *BRCA1/2* mutations could have prognostic and predictive significance among ovarian cancer patients. First of all, it has been suggested that the site of *BRCA* mutation is associated with relative susceptibility to ovarian cancer, underlying a genotype–phenotype correlation. Characteristically, mutations located within the 3′ portion of *BRCA1* have long shown to be less likely to lead to OC compared to the ones found in the 5′ end of the gene [19,20,21]. Analogously, truncating *BRCA2* mutations that lie within the Ovarian Cancer Cluster Region (OCCR) of exon 11 (nucleotides 3035-6629) have been correlated with significantly higher risk of OC [22,23,24]. These mutations lie within the RAD51-binding domain (RAD51-BD) of the *BRCA2* gene; ovarian cancer patients harboring mutations located at the RAD51-BD (exon 11) of the *BRCA2* gene have prolonged survival compared to mutations occurring at other locations of the *BRCA2* gene or to noncarriers [25]. Despite not detecting any strong correlations between *BRCA1* mutation location and survival outcome, we found that 45.7% (16/35) of *BRCA1* mutations were located towards the 5′ end of the gene and are known to target the highly conserved C-terminal BRCT repeats that function as a phosphoprotein-binding domain. In addition, differences in survival outcomes according to the *BRCA2* mutation position were not observed in our study. When comparing our results to those of older studies, it must be pointed out that there are also multiple reports that showed no significant differences on OC risk associated with specific BRCA2 regions [26,27], in accordance with our findings. The conflicting findings reported in the literature as well as the different survival outcomes of carriers of the same mutations found in this study might indicate the presence of additional factors that modify the risk. Genetic factors such as TP53 mutations [28] and clinical factors including disease extent that determines initial therapeutic approach [29] are known to affect survival irrespective of the presence of *BRCA1/2* mutations. Whether *BRCA1/2* mutation types may affect disease extent at diagnosis, however, remains currently elusive.

Interestingly, we observed noteworthy differences, in terms of PFS duration, between carriers of identical mutations in the same *BRCA* gene, as is shown with bars of the same color in the graph of Figure 3. These dissimilarities in outcome further substantiate the notion that the clinical course of ovarian cancer is not solely determined by mutation type/location. Especially in the case of HGSOC, recent evidence suggests that there are several other genetic abnormalities and co-contributing factors that determine the biology and extent of the disease [30]. Specifically, about half of HGSOC cases are characterized by homologous recombination repair deficiency (HRD) due to chromosomal instability, which is caused by a multitude of additional genetic abnormalities, apart from *BRCA1/2* mutations [31]. From this perspective, HRD score has been used to acquire information regarding the magnitude of the potential treatment benefit of niraparib, a PARP inhibitor [9].

The presence of a deleterious *BRCA* mutation has been shown to affect survival not only in primary ovarian carcinoma but even in the fairly common recurrent setting, with 75% of HGSOC patients relapsing within two years of diagnosis [32,33]. Advances in current interventions, including cytoreductive surgery, and the incorporation of targeted therapy in the chemotherapeutic armamentarium, seem to collectively impact progression-free survival [34,35]. Characteristically, PARP (poly (ADP-ribose) polymerase) inhibitors lead to the accumulation of DNA single strand breaks (SSBs), inducing the formation of double strand breaks (DSBs); thus, they are known for their effectiveness against tumors characterized by faulty homologous repair mechanisms (HRD), such as *BRCA*-mutated tumors [36]. In the context of this work, we noted that OC patients treated with PARP inhibitors exhibit similar survival outcomes, independently to the *BRCA1/2* mutation location. Yet, according to the literature, tumors carrying pathogenic mutations that affect the RING domain of BRCA1 have been shown to respond poorly to PARP inhibition and rapidly develop resistance, due to the reduction of BRCA1/BARD1 heterodimerization and the inhibition of its ubiquitin ligase function [37]. Therefore, determining how a specific mutation affects the structure of BRCA1/2 has potential implications for the evaluation of existing treatment options. However, in our cohort, we detected only three patients with BRCA1 mutations affecting this specific region of the gene, possibly implying that sample size may have contributed to a lack of statistical significance when stratifying the survival analyses by mutation position.

Certain limitations of this study should be acknowledged, such as small sample size that might undervalue the exact impact of the mutation site on survival outcome, rendering our results merely indicative and in need of further confirmation in a larger cohort of patients. Furthermore, it is important to point out that in our study cohort were only included women with germline *BRCA1/2* mutations due to limited availability of tissue testing during the period of diagnosis. It is known that in certain cases presence of germline *BRCA1/2* mutations may not be related to tissue carcinogenesis either due to persistence of the wild-type allele (no Loss of Heterozygosity) [38] or due to reversing mutations in the tumor [39]. Therefore, accumulating data from ovarian cancer patients who undergo full gene sequencing in both tissue and blood will eventually strengthen our understanding of the precise effect of specific rearrangements and mutations on survival outcome.

## 4. Materials and Methods

### 4.1. Patients

We retrospectively searched our institutional database for patients with advanced stage (II-IV) high-grade serous carcinoma that tested positive for germline mutations of *BRCA1* and *BRCA2* genes, during a 5-year period (2015–2019). Informed consent was obtained from all patients involved in the study, with respect to their treatment regimen, genetic testing procedure, as well as granting access to their medical records for the purposes of data acquisition. The clinicopathological characteristics that were acquired from medical reports included age at diagnosis, stage, surgical management, *BRCA1/2* mutation status, targeted therapy, progression of the disease, and overall survival. The present study received approval from the Institutional Review Board (IRB) of Alexandra Hospital in Athens, Greece and was carried out in conformity with the ethical guidelines of the Declaration of Helsinki.

### 4.2. Genetic Analysis

**Figure 4:**
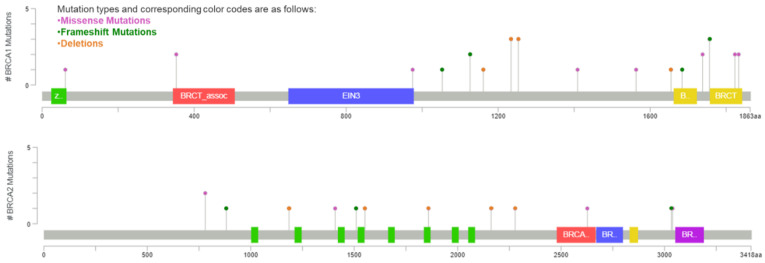
Germline mutations of BRCA1 and BRCA2 genes in the study cohort of ovarian cancer patients (whole exon deletions are not depicted). Mutation diagram circles are colored with respect to the corresponding mutation types. Abbreviations: zf-C3HC4: zinc finger, C3HC4 type (RING finger) (aa 24–64), BRCT_assoc: serine-rich domain associated with BRCT (aa 344–507), EIN3: ethylene insensitive 3 (aa 648–978), BRCT: BRCA1 C Terminus (BRCT) domain (aa 1757–1842), BRCA-2_helical: BRCA2, helical (aa 2479–2667), BRCA-2_OB1: BRCA2, oligonucleotide/oligosaccharide-binding, domain 1 (aa 2670–2799), BRCA-2_OB3: BRCA2, oligonucleotide/oligosaccharide-binding, domain 3 (aa 3052–3190).

Blood samples were collected from each participant for germline DNA testing when the patients were referred to a Genetic Counseling Unit. All participants were screened for *BRCA1* and *BRCA2* mutations, either by Sanger sequencing using Applied Biosystems’ 3130XL Genetic Analyzer (Thermo Fisher Scientific, Carlsbad, CA, USA) or by massive parallel sequencing using the TruSight Cancer panel (Illumina, San Diego, CA, USA) on a MiSeq Analyzer, as previously described [40]. Carriers of variants of uncertain/unknown significance (VUSs) were deemed as non-carriers. The NM_007294/ENST00000357654 and NM_000059/ENST00000380152 were used as the reference sequences for *BRCA1* and *BRCA2* genes, respectively. According to the typical BRCA exon numbering used, the *BRCA1* gene has 24 exons and encodes 1863 amino acids, while *BRCA2* consists of 27 exons and encodes 3418 amino acids (Figure 4).

### 4.3. Statistical Analysis

Continuous variables were summarized by using descriptive statistical measures (median and percentiles (25th, 75th)). Additionally, categorical variables were displayed as percentages with the use of frequency tables (N, %). Overall Survival (OS) was defined as the time interval from initiation of chemotherapy until death from any cause. Progression-free Survival (PFS) was defined as the time between the start of chemotherapy and the date of progression. Alive patients were censored at the date they were last conducted. In order to describe and visualize the effect of categorical variables on OS and PFS, Kaplan–Meier estimates were utilized. Notably, log-rank tests were used to assess the prognostic value of categorical variables on clinical outcomes. These factors were correlated with PFS and OS according to hazard ratios and their 95% confidence intervals estimated from univariate Cox proportional hazards models. All statistical analyses were performed using the SPSS software.

## 5. Conclusions

In conclusion, this study offers an initial assessment of genotype–phenotype interplay in *BRCA*-mutated ovarian cancer, based on mutation location analysis and its effect on survival outcome. Future studies examining the type of mutation in combination with mutation site within the gene could offer further insight into the multifaceted issue of ovarian cancer prognosis. In addition, further investigations exploring the genotype–phenotype correlations of specific *BRCA1* and *BRCA2* mutations that differentiate prognosis among family members who are carriers of the same mutations are needed to clarify the role of other genetic abnormalities and co-contributing risk factors in the clinical course of *BRCA*-mutated ovarian cancer patients.

## Figures and Tables

**Table 1:** Clinical, genetic, and treatment characteristics of *BRCA1/2*-mutated serous ovarian cancer patients.

Characteristic		
	Median (25th–75th Perc)	Missing (%)
**Age**	54.2 (45.8–62.1)	0 (0)
	*n*	**(%)**
**Stage**		
**II**	8	15.7%
**IIIA/IIIB**	4	7.9%
**IIIC**	29	56.9%
**IV**	10	19.6%
**Debulking Surgery**		
**Primary**	31	60.8%
**Interval**	20	39.2%
**Surgical Outcome**		
**Complete**	45	88.2%
**Optimal/Suboptimal**	6	11.8%
**Mutated Gene**		
***BRCA1***	35	68.6%
***BRCA2***	16	31.4%
**Type of mutation**		
**Deletion**	26	51.0%
**Frameshift**	4	7.8%
**Insertion**	4	7.8%
**Missense**	17	33.3%
**Affected region**		
**RING domain**	3	5.9%
**BRCA1 exons 11–13**	16	31.4%
**BRCT domain**	16	31.4%
**RAD51 binding domain**	11	21.6%
**DNA binding domain**	5	9.8%
**Bevacizumab administration**		
**No**	31	60.8%
**Yes**	20	39.2%
**PARP inhibitor administration**		
**No**	17	33.3%
**Yes**	34	66.7%

**Table 2:** Median PFS, OS with 95% CI, Univariate analysis.

		PFS			OS	
	Median	95% CI	*p*-Value *	Median	95% CI	*p*-Value
**Age**			0.867			0.467
**≤65**	23.9	18.4–29.3		92.9	59.8–126.1	
**>65**	23.0	20.3–25.7		NR	NR	
**Stage**			0.155			0.173
**II/IIIA/IIIB**	45.8	NR		NR	NR	
**IIIC/IV**	21.7	13.9–29.5		81.9	63.6–100.2	
**Mutant gene**			0.828			0.405
***BRCA1***	23.0	19.1–26.9		81.9	-	
***BRCA2***	23.8	16.3–31.3		92.9	32.9–153.7	
**Surgical outcome**			**<0.001**			**0.028**
**Complete**	24.9	18.4–31.4		92.9	69.3–116.7	
**Optimal/Suboptimal**	11.1	2.5–19.8		45.2	17.4–72.8	
**Surgical outcome**			0.459			0.178
**Primary**	23.0	12.1–33.9		NR	NR	
**Interval**	23.9	19.9–27.8		81.9	36.7–127.1	
**Type of mutation**			0.369			0.187
**Deletion**	19.7	13.4–26.1		92.9	40.8–145.2	
**Frameshift**	30.1	21.1–39.1		NR	NR	
**Missense**	23.0	18.1–27.9		NR	NR	
**Affected domain**			0.339			0.882
**RING domain**	15.6	-		77.1	10.9-NR	
**BRCA1 exons 11–13**	21.8	18.1–25.5		81.9	-	
**BRCT domain**	28.7	19.9–37.6		NR	NR	
**DNA Binding domain**	19.7	12.9–26.6		NR	NR	
**RAD51 binding domain**	33.9	16.0–51.9		92.9	25.9–160.1	
**Bevacizumab**			0.707			0.214
**No**	23.9	19.6–28.2		NR	NR	
**Yes**	21.8	6.6–36.9		81.9	69.8–116.1	

* Log-rank test; NR = not reached.

## Data Availability

No new data were created or analyzed in this study. Data sharing is not applicable to this article.

## Supplementary Materials

The following are available online at https://www.mdpi.com/article/10.3390/curroncol28060377/s1, Figure S1: Pairwise comparisons of PFS and OS between different functional domains of BRCA1 (A,B) and BRCA2 (C,D) gene.
