# Supplementary material for: BRCA1/2 Mutation Types Do Not Affect Prognosis in Ovarian Cancer Patients
Source: Curr Oncol. 2021 Nov 3;28(6):4446–56. doi: 10.3390/curroncol28060377 (PMC8628789; doi:10.3390/curroncol28060377)

Current Oncology

Article

BRCA1/2 Mutation Types Do Not Affect Prognosis in Ovarian Cancer Patients

Michalis Liontos, Eleni Zografos, Panagiotis Zoumpourlis, Angeliki Andrikopoulou, Anna Svarna, Oraianthi Fiste, Elena Kunadis, Alkistis Maria Papatheodoridi, Maria Kaparelou, Konstantinos Koutsoukos, Nikoloas Thomakos, Dimitrios Haidopoulos, Alexandros Rodolakis, Meletios-Athanasios Dimopoulos and Flora Zagouri

Figure S1. Pairwise comparisons of PFS and OS between different functional domains of BRCA1 (**A**,**B**) and BRCA2 (**C**,**D**) gene.

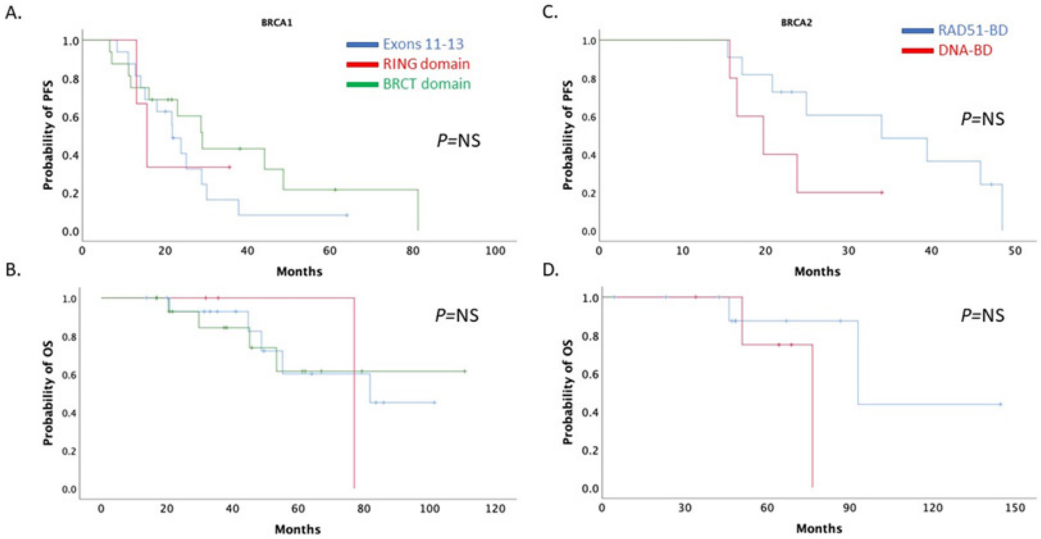

Supplement: Supplementary file 1 [file curroncol-28-00377-s001.zip › curroncol-1431002-supplementary-final .pdf]
